# Transcriptome analysis of dominant-negative Brd4 mutants identifies Brd4-specific target genes of small molecule inhibitor JQ1

**DOI:** 10.1038/s41598-017-01943-6

**Published:** 2017-05-10

**Authors:** Tim-Michael Decker, Michael Kluge, Stefan Krebs, Nilay Shah, Helmut Blum, Caroline C. Friedel, Dirk Eick

**Affiliations:** 1 Department of Molecular Epigenetics, Helmholtz Center Munich and Center for Integrated Protein Science Munich (CIPSM), Marchioninistrasse 25, 81377 Munich, Germany; 2 Institute for Informatics, Ludwig-Maximilians-Universität München, Amalienstr. 17, Munich, 80333 Germany; 3 Laboratory for Functional Genome Analysis (LAFUGA) at the Gene Center, Ludwig-Maximilians-Universität München, Munich, Germany

## Abstract

The bromodomain protein Brd4 is an epigenetic reader and plays a critical role in the development and maintenance of leukemia. Brd4 binds to acetylated histone tails and activates transcription by recruiting the positive elongation factor P-TEFb. Small molecule inhibitor JQ1 competitively binds the bromodomains of Brd4 and displaces the protein from acetylated histones. However, it remains unclear whether genes targeted by JQ1 are mainly regulated by Brd4 or by other bromodomain proteins such as Brd2 and Brd3. Here, we describe anti-proliferative dominant-negative Brd4 mutants that compete with the function of distinct Brd4 domains. We used these Brd4 mutants to compare the Brd4-specific transcriptome with the transcriptome of JQ1-treated cells. We found that most JQ1-regulated genes are also regulated by dominant-negative Brd4 mutants, including the mutant that competes with the P-TEFb recruitment function of Brd4. Importantly, JQ1 and dominant-negative Brd4 mutants regulated the same set of target genes of c-Myc, a key regulator of the JQ1 response in leukemia cells. Our results suggest that Brd4 mediates most of the anti-cancer effects of JQ1 and that the major function of Brd4 in this process is the recruitment of P-TEFb. In summary, our studies define the molecular targets of JQ1 in more detail.

## Introduction

The discovery of epigenetic mechanisms has advanced our understanding of gene regulation. Intriguingly, the regulation of oncogene expression by epigenetic mechanisms has inspired the development of new strategies for cancer therapy^1^. Acetylation of histone tails is mostly associated with upregulation of gene expression^2^. Proteins that write and erase these modifications have been studied initially, while proteins that read and interpret histone acetylation have caught the attention of researchers and clinicians only recently. Specifically, bromodomain protein Brd4 is a ‘reader’ protein that is currently evaluated as a potential drug target in cancer therapy.

Brd4 belongs to the bromodomain and extra-terminal (BET) protein family which includes three somatic members Brd2, Brd3, Brd4, and the testis-specific Brd-t^3, 4^. The N-terminus of all BET proteins comprises two bromodomains, BD1 and BD2, followed by an extra-terminal ET domain. BET proteins are recruited to active chromatin by binding to acetylated histone tails via the two bromodomains^3, 5^. Notably, Brd4 has a unique C-terminal domain that binds positive transcription elongation factor b (P-TEFb). P-TEFb is essential for the release of paused RNA polymerase II, which is a crucial regulatory step within the transcription cycle^6–8^. Brd4 further binds to super-enhancers, a class of highly active enhancers that define cell identity and regulate oncogenic drivers in several tumor cell lines^9, 10^.

Functional analysis of Brd4 and its evaluation as a drug target was boosted by the discovery of BET-inhibitors. This class of drugs competitively binds to the bromodomains of BET proteins and blocks the interaction with acetylated histone tails. Starting from 2010, when two inhibitors of BET proteins, JQ1 and I-BET, were reported^11, 12^, the therapeutic potential of BET inhibitors was recognized, and several compounds are studied in ongoing clinical trials^13^. BET inhibitors suppress key oncogenes in many cancer cell lines. For instance, JQ1 potently suppressed expression of c-Myc and c-Myc-regulated genes in multiple myeloma and Burkitt lymphoma cells^14^, as well as in a mouse model for acute myeloid leukemia^15^. JQ1 lead to broad eviction of Brd4 from chromatin in B-cell tumors and repressed genes were enriched for c-Myc and E2F targets^16^. Similar results were reported for I-BET, which inhibited transcription of *BCL2*, *c-Myc* and *CDK6* in promyelocytic leukemia cells^17^. Together these studies suggest a mechanism for BET inhibitors that specifically represses genes essential for cell proliferation.

Available inhibitors target all four BET proteins, Brd2, Brd3, Brd4, and Brd-t^11, 12^ and hence cannot be used to study a specific protein alone. While many studies focus on Brd4, less is known about other BET family members and their response to BET inhibitors. Interestingly, Brd2 and Brd3 have also been reported to play important roles in the development and maintenance of human cancers^13, 18^. Thus, it remains unclear whether Brd4 is indeed the protein that mediates the anti-tumor effects of BET inhibitors. Unfortunately, bromodomain-independent functions of Brd4 cannot be studied by BET inhibitors. Therefore, we aimed to study Brd4 functions using a different approach.

Here, we coexpressed fragments of Brd4 to target the function of the endogenous protein in a domain-specific way, a strategy also known as dominant-negative inhibition. By means of RNA-seq we found most of the genes that were differentially expressed upon JQ1 treatment also differentially expressed upon dominant-negative inhibition of Brd4. Furthermore, expression of the P-TEFb-interacting domain of Brd4 changed gene expression in a similar way as targeting the bromodomain function alone. Our findings demonstrate that transcriptomic changes induced by the BET inhibitor JQ1 are also mediated by dominant-negative Brd4 mutants further indicating that the function of the bromodomains is directly linked to the ability of Brd4 to recruit P-TEFb.

## Results

### Establishment of an inducible expression system for Brd4 mutants

**Figure 1:**
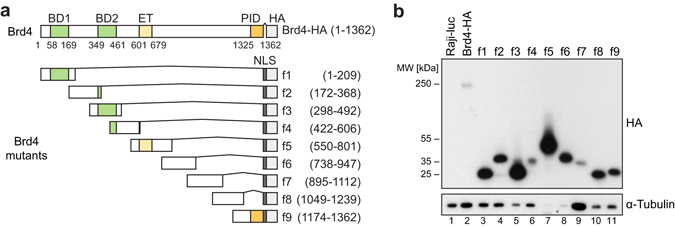
Expression system for Brd4 mutants. (**a**) Schematic structure of recombinant full-length human Brd4 protein (Brd4-HA) and Brd4 fragments f1–f9. BD1/2 = bromodomain 1/2, ET = extra-terminal domain, PID = P-TEFb-interacting domain, HA = hemagglutinin tag, NLS = nuclear localization signal. (**b**) Expression levels of HA-tagged Brd4 mutants, assessed via western analysis of the HA tag. α-Tubulin served as loading control. Samples were diluted with loading buffer prior to loading to visualize all signals on a single membrane (dilution ratios: lanes 2 and 9, 1:1; lanes 1, 3, 4, 6, 10 and 11, 1:10; lanes 5, 7 and 8, 1:20). Raji-luc cells expressed an non-tagged luciferase and served as negative control.

To inhibit the function of Brd4 in a domain-specific way, we constructed dominant-negative mutants. To this end, full length Brd4 was divided into 9 overlapping fragments (f1–f9) of about 200 amino acids in length (Fig. 1a). The constructs were cloned into the pRTS vector, which contains a bidirectional promoter allowing simultaneous expression of the Brd4 fragment and eGFP^19^. eGFP was used as a reporter for efficient induction. Vectors were stably transfected into Burkitt lymphoma Raji cells, and cells were selected with hygromycin B. Expression was induced by doxycycline. Western analysis revealed that all Brd4 fragments f1–f9 were properly expressed, although at different levels (Fig. 1b). Full-length Brd4 (Brd4-HA) and f7 yielded low signals compared to f1, f2, f4, f8 and f9 with intermediate and f3, f5 and f6 with high levels of expression. No HA signal was detected in cells transfected with a luciferase-expressing construct (Raji-luc), which served as a control.

### Expression of Brd4 fragments inhibits cell proliferation

**Figure 2:**
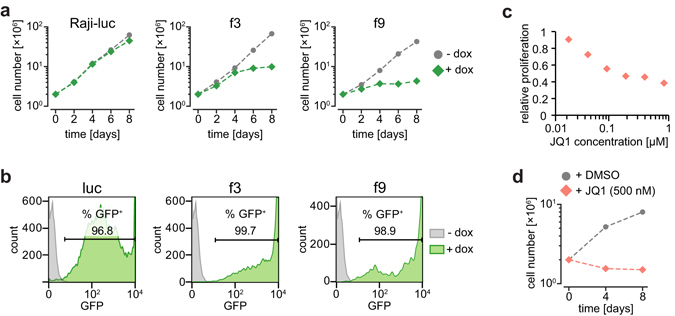
Proliferation assay of Raji cells expressing f3 and f9 or after treatment with JQ1. (**a**) Non-induced (gray) and induced cells (25 ng/ml doxycycline, green) were plotted. Induced cells expressed either luciferase (Raji-luc, negative control) or dominant-negative Brd4 mutants f3 and f9. (**b**) Percentage of induced cells (green) were assessed by measuring GFP reporter signals using flow cytometry. Uninduced Raji f2 cells served as negative control (gray). (**c**) Raji cells were treated with increasing concentrations of JQ1 for 72 hours. Cell proliferation was measured via MTS assay and plotted against JQ1 concentration (log scale). (**d**) Induced Raji-luc cells were treated with 500 nM JQ1 (red)/DMSO (gray, control) for 8 days. Living cell numbers were counted and plotted against time.

We next investigated if overexpression of specific Brd4 domains can inhibit cell proliferation. For this purpose, we screened all cloned Brd4 constructs for a dominant-negative phenotype using proliferation assays. After induction of the Brd4 constructs, cell proliferation was monitored for 8 days. Two days after induction we consistently detected that proliferation rates were (i) substantially reduced for cells expressing Brd4 fragments f3, f5, f9, (ii) intermediately reduced for f4 and f6, and (iii) not affected in the control cell line Raji-luc or other Brd4 fragments (Fig. 2a and Supplementary Fig. 1). Proliferation rates were measured in three biological replicates. To verify efficient induction of the constructs in all cells, a prerequisite for dominant-negative inhibition, we measured GFP-reporter signals on day 8 via flow cytometry and found induction rates of ~90% for most samples (Fig. 2b and Supplementary Fig. 2). Notably, we could confirm the dominant-negative phenotype of Brd4 mutants in the non-small cell lung carcinoma cell line H1299 (Supplementary Fig. 3). We considered mutants f3 (inhibition of bromodomain function) and f9 (inhibition of P-TEFb-interacting domain function) as most interesting dominant-negative mutants for inhibition of Brd4 function (dnBrd4). In the following experiments the impact of these two mutants on the cellular transcriptome of Raji cells was compared with the impact of JQ1.

### JQ1 inhibits cell proliferation at nano-molar concentrations

We first determined the concentration of JQ1 necessary for growth inhibition of Raji cells. We found that 100 nM JQ1 inhibited cell proliferation of wild-type Raji cells by 50% after 72 h of treatment (Fig. 2c). Next, we tested the long-term effects of JQ1 on Raji-luc cells, which served as control cell line in the subsequent experiments. We detected complete inhibition of cell proliferation with 500 nM JQ1 (Fig. 2d). Thus, we considered 500 nM JQ1 as suitable concentration for the following transcriptome analysis.

### Preparation and quality control of RNA-seq libraries

To characterize genes that are regulated by dnBrd4 mutants and to define the overlap between JQ1-regulated genes and dnBrd4-regulated genes, we performed RNA-seq. We prepared libraries of poly-A enriched RNAs of five biological replicates for f3 and f9 dominant-negative Brd4 mutants, JQ1-treated Raji-luc cells and DMSO-treated Raji-luc cells as control. All libraries were prepared 24 hours after inducing the dnBrd4 mutants or 24 hours after adding JQ1. In parallel, cells were tested for sufficient induction, as determined by eGFP expression (Supplementary Fig. 4).

**Figure 3:**
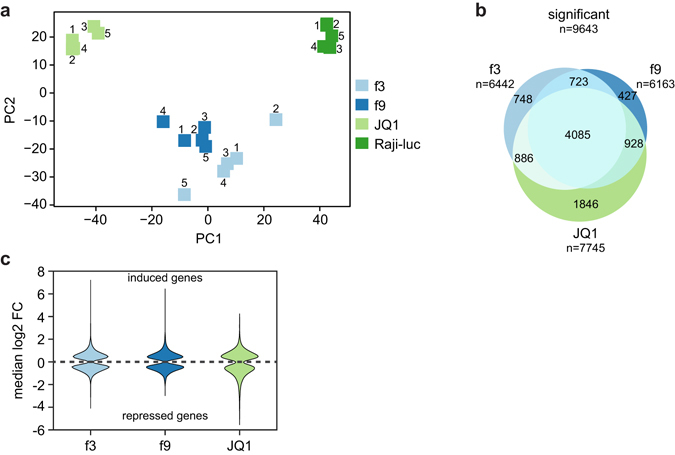
Analysis of transcriptomes JQ1-treated Raji cells and dnBrd4 mutants f3 and f9. (**a**) Principal component analysis of 20 RNA-seq samples; 5 replicates per condition. f3 replicate no. 2 did not cluster properly with the other f3 replicates and was excluded from further analysis (see also Supplemental Fig. 5). (**b**) Significantly (p-value: ≤0.05) differentially expressed genes of f3, f9 and JQ1-treated Raji cells were plotted as Venn diagram to visualize commonly regulated genes. (**c**) Distribution of median log2 fold-change for DE genes. Induced genes were plotted above the dashed line, reduced genes below. Median log2 FCs were calculated using the log 2 FC values determined by the differential expression programs that found a statistical significant change (at least 2 out of limma, edgeR, DEseq2).

As quality control for low variation among the five biological replicates, we performed principal component analysis (PCA). Four clearly separated groups of samples were identified by the PCA (Fig. 3a), corresponding to the four investigated conditions. Here, replicates of the same sample clustered closely together and were simultaneously separated from other conditions (Fig. 3a). Furthermore, replicates of f3 and f9 clustered in close proximity, suggesting higher similarities of their transcriptomes compared to the replicates of JQ1, which were more distant to all other conditions in our analysis. Higher variation was observed for replicates 2 and 5 of condition f3 (f3–2 and f3–5), which did not cluster as closely with the rest of the f3 replicates. Similar results were obtained when performing hierarchical clustering analysis based on the Euclidean distance (Supplementary Fig. 5). Here, f3–5 clustered with the remaining f3 replicates, while f3–2 clustered close to the DMSO-treated Raji-luc control group. Thus, replicate f3–2 was excluded from further analysis. In summary, this demonstrated high quality of our RNA-seq datasets, allowing for an in-depth differential expression analysis.

### JQ1 and dnBrd4 mutant f3 regulate a common set of genes

Next we asked how JQ1 treatment and expression of dnBrd4 in mutant f3 affect the transcriptome. To this end, we performed differential expression analysis relative to the DMSO-treated Raji-luc control and calculated the overlap of differently expressed (DE) genes (p-value ≤ 0.05, no fold-change cutoff). Remarkably, 66% and 55% of expressed genes were significantly differentially expressed under JQ1 treatment and expression of the f3 mutant, respectively, indicating substantial deregulation of gene expression. More importantly, 4971 of 7745 JQ1-regulated genes (64%) were also regulated by f3 (Fig. 3b). This suggests that Brd4 fragment f3 comprising BD2 potentially inhibits Brd4 similar to JQ1 by blocking the bromodomain function. Importantly, the direction of the change in gene expression was the same for DE genes of JQ1 and f3 conditions: 95% of the common DE genes were consistently repressed (2395) or induced (2321) by both f3 and JQ1 (Supplementary Fig. 6a) and the rank correlation between corresponding fold-changes was 0.84. Thus, JQ1 and dnBrd4 f3 regulate the transcriptome of Raji cells in a very similar manner with the same set of genes being either repressed or induced.

### Brd4 PID is linked to bromodomain function

Since both, JQ1 and f3, inhibit the bromodomain function of Brd4, we further investigated the consequences of inhibiting the P-TEFb interacting PID domain of Brd4. For this purpose we compared the transcriptomes of dnBrd4 mutants f9 and f3. Strikingly, f9 DE genes accounted for 4808 of f3 DE genes (75%) (Fig. 3b) and rank correlation of significant fold-changes was 0.93. This demonstrates that dnBrd4 mutants f3 and f9 regulate a large set of common genes in a similar way, and further suggests that the gene regulatory activity of Brd4 requires the PID domain. Next we compared the transcriptomes of f9 and JQ1-treated cells. Here, f9 DE genes accounted for 5013 DE genes of JQ1-treated cells (65%) with a rank correlation of fold-changes of 0.9. This suggests that disruption of the Brd4 PID function also has similar effects on the transcriptome as JQ1. In summary, inhibition of BD2 and PID domains of Brd4 caused strongly overlapping changes in gene expression, suggesting that both domains are functionally linked.

### JQ1 deregulates gene expression more strongly than dnBrd4 mutants

Interestingly, comparison of median fold-changes of regulated genes between conditions indicated that the effect of JQ1 on gene expression was more pronounced than for the dnBrd4 mutants. While median fold-changes for either up- or down-regulated genes were ~1.46 for the dnBrd4 mutants, under JQ1 treatment they were ~1.52 for up-regulated genes and 1.67 for down-regulated genes. These differences are small, but are highly statistically significant (Wilcoxon rank sum test, p-value < 10^−5^) and have a considerably effect on the number of DE genes identified at different fold-change cut-offs. If we successively applied ≥1.33-fold, ≥1.5-fold and ≥2-fold-change filters to our dataset of DE genes, the resulting Venn diagrams revealed that more JQ1-regulated genes (1949 at fold-change 2) passed these increasing filters compared to genes regulated by mutants f3 and f9 (811 and 759, respectively, Supplementary Fig. 6b). This indicates that JQ1 affects gene expression more strongly than dnBrd4 mutants f3 and f9.

### Genes activated by Brd4 inhibition

Many studies on transcriptomic changes induced by BET inhibitors focus on downregulation of oncogenes and thereby highlight the activating function of Brd4. However, the role of BET proteins as transcriptional repressors is well known^4^. To investigate the repressive role of Brd4, we compared up- and downregulated genes. In all three groups, JQ1, f3, and f9, around 50% of genes were upregulated (Fig. 3c), suggesting that the nuclear function of Brd4 is not limited to activating transcription but it also includes the repression of many genes. This repression of various genes is not necessarily a direct transcriptional repression by Brd4, but likely a downstream effect resulting from Brd4-mediated upregulation of transcriptional repressors. For instance, cyclin dependent kinase inhibitors *CDKN1B* and *CDKN2B*, which are significantly upregulated by f3, f9 and JQ1 (1.59-fold to 3.7-fold) (Supplementary Table 2a), have been shown to be transcriptionally repressed by c-Myc^20^. Another interesting set of genes upregulated by f3, f9 and JQ1 are *AFF1*, *AFF4* and *AF9*, members of the super-elongation complex (SEC) (Supplementary Table 2b). Since SEC, like Brd4, binds P-TEFb^21, 22^, this suggests a balancing mechanism in which SEC is upregulated upon inhibition of Brd4. These regulatory circuits further exemplify the complex role of Brd4 in transcriptional gene regulation.

### Brd4 DE genes are enriched for c-Myc target genes

**Figure 4:**
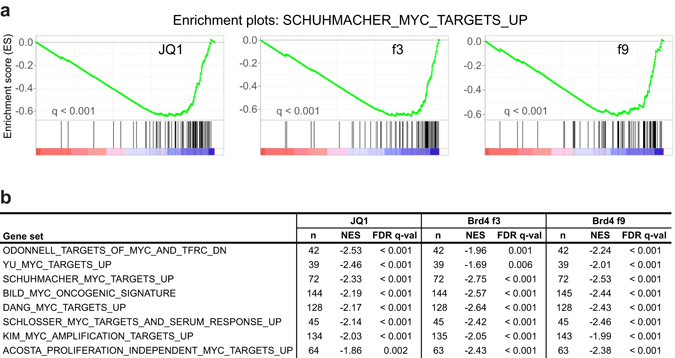
Gene set enrichment analysis (GSEA) of c-Myc target genes. (**a**) C-Myc signature enrichment plots in Raji-luc + 500 nM JQ1, Brd4 f3, and Brd4 f9 cells versus DMSO-treated Raji-luc cells as a control for all three conditions. Plots were prepared using the SCHUHMACHER_MYC_TARGETS_UP^24^ dataset (containing genes upregulated by c-Myc) available at the Molecular Signatures Database^51^. C-Myc upregulated target genes were enriched at the bottom of the ranked list of genes (ranking according to log2 fold-changes obtained from the previous DE analysis), indicating that genes upregulated by c-Myc tended to be repressed. (**b**) Table of selected c-Myc target gene sets^24–31^ enriched in all three samples. n = number of genes in each set; NES = normalized enrichment score; FDR q-val = test of statistical significance. Negative NES indicates that genes in the respective target gene sets tended to be repressed.

C-Myc regulated genes are well known downstream targets of JQ1 and are described as main mediators of the anti-proliferative effects of BET inhibition^14, 23^. We tested if c-Myc target genes are enriched in f3 and f9 DE genes in a comparable manner as JQ1 DE genes. To this end we performed gene set enrichment analysis (GSEA) using ranked lists of JQ1, f3, and f9 DE genes. We detected significant (FDR q-val < 0.001) enrichment of genes upregulated by c-Myc at the bottom of the ranked list, demonstrating that these genes tended to be similarly repressed in all three conditions (Fig. 4a). Importantly, the enrichment for c-Myc target genes was robustly reproduced for numerous other available gene sets (Fig. 4b)^24–31^. In summary, the inhibition of proliferation observed in f3 and f9 mutant cell lines is likely to be mediated by repressing c-Myc and its target genes. This further illustrates that the mechanisms of dominant-negative inhibition of Brd4 and BET inhibition using JQ1 largely overlap.

## Discussion

The individual contribution of BET proteins Brd2, Brd3, Brd4 and Brd-t to the anti-proliferative effects of JQ1 still remains unclear. In this study, we investigated the Brd4-specific target genes of JQ1. We constructed and analyzed dominant-negative mutants of Brd4 and determined if these mutants can induce changes in gene expression similar to JQ1. In our studies we focused on the mutants f3 and f9 as expression of both mutants strongly inhibited proliferation of Raji and H1299 cells. Mutant f3 expresses bromodomain 2 (BD2), which competes with the binding of endogenous Brd4 to acetylated histone tails, as previously observed for a double-bromodomain inhibitor (BD1/BD2)^32^. Comparison of the transcriptome of mutant f3 with the transcriptome of JQ1-treated cells revealed major changes in gene expression in both transcriptomes and that the dominant-negative mutant f3 repressed and induced almost the same genes as JQ1. Thus, mutant f3 confirms the current model that JQ1 may act mainly by inhibiting the interaction of the bromodomains of Brd4 and other BET proteins with actetylated histone tails. We further noticed that JQ1 induced higher changes in gene expression in affected genes than the f3 mutant. Previous work showed that the target genes of Brd2, Brd3, and Brd4 partially overlap as demonstrated for cytokine genes after siRNA-mediated knockdown of BET proteins^33^. Thus, higher JQ1-induced changes in gene expression may be explained by additional inhibition of Brd2 and Brd3.

Mutant f9 expresses the P-TEFb-interacting domain (PID) and thereby inhibits the recruitment of P-TEFb to Brd4 and acetylated chromatin. The inhibitory effect on P-TEFb recruitment of PID expression or of synthetic peptides containing PID has been reported before^32, 34^. We found that changes induced in the transcriptome of mutant f9 largely overlapped with the changes that we observed for mutant f3 and JQ1-treated cells. In fact, the functional overlap of f9 with f3 and JQ1 was confirmed by an almost identical impact of all three inhibitors on the expression on c-Myc target genes. This result was not necessarily expected, because the contribution of the PID domain to the gene regulatory function of Brd4 has not been measured quantitatively before. The direct comparison of mutant f3 and f9 in our study demonstrates that BD2 and PID in Brd4 act as a functional unit and that the gene regulation by Brd4 largely depends on PID. However, it remains unclear whether the PID does interact only with P-TEFb or in addition with other factors. Here we found that a large set of genes activated by JQ1 and mutant f3 is found activated also by mutant f9. This may be due to downstream effects that are triggered by genes regulated by P-TEFb or further exemplifies the role of Brd4 as a transcriptional repressor. Together, our results suggest that gene regulatory changes induced by JQ1 can also be induced by dominant-negative Brd4 mutants, and that the inhibition of the Brd4 PID domain is central for the function of BET-inhibitor JQ1.

The functional link observed between PID and bromodomains of Brd4 raises the question how the short isoform of Brd4 (Brd4-s), which lacks the PID^34^, is involved in transcriptional regulation and if inhibition of Brd4-s by JQ1 may also translate into a phenotype. Brd4-s might act as a dominant-negative inhibitor of full-length Brd4, as suggested by previous studies^35, 36^. However, differential biochemical and nuclear localization properties indicate that the two isoforms of Brd4 may fulfill separate roles. Brd4-s localizes specifically to the transcriptionally inactive perinuclear region where it might be involved in gene repression^37^.

Our results further establish that Brd4 inhibition results in both activation and repression of genes to a similar extent. Although activation of genes by inhibition of BET proteins may to some extent be a downstream effect, the function of BET proteins as transcriptional repressors has been reported before, including the original I-BET report^12^. Brd2 forms nuclear complexes with Swi/Snf chromatin remodelers that co-activate and co-repress transcription^38^. Furthermore, a recent report demonstrated the repressive role of BET proteins for the transcriptional coactivator TAZ^39^. We observed that *AFF1*, *AFF2* and *AF9*, encoding three major subunits of the super elongation complex (SEC), were upregulated by JQ1 as well as in mutants f3 and f9. SEC can also bind P-TEFb^21^, suggesting a feedback loop and a crosstalk between Brd4 and SEC. In line with this, a recent model proposes recruitment of P-TEFb by Brd4 and SEC via different mechanisms^22^. The upregulation of SEC might be a response to Brd4 inhibition to rescue RNA polymerase II (Pol II) elongation, in agreement with a recent study that demonstrated compensatory Pol II loading at JQ1-insensitive genes^16^. The observation that many genes are upregulated upon inhibition of Brd4 possibly results from a combination of direct and indirect mechanisms.

The PID domain is present in Brd4 but absent in Brd2 and Brd3. Nevertheless, the dominant-negative mutant f3 might also compete with the binding of Brd2 and Brd3 bromodomains to acetylated histone tails, and may thereby affect the function of Brd2 and Brd3 as well. However, our transcriptome analyses revealed that most of the gene regulatory function of Brd4 is linked to the PID domain. Thus it seems unlikely that inhibition of Brd2 and Brd3 can induce transcriptional changes in a similar range. Besides functional redundancy in some cases, Brd2, Brd3 and Brd4, may have individual roles, as shown for the regulation of metabolic pathways in the pancreatic β cell^40^. Moreover, it was recently observed that Brd2 and Brd4 co-regulate transcription by exerting discrete functions in mouse Th17 cell differentiation^41^. Therefore, dominant-negative inhibitor approaches directed against Brd2 and Brd3 are necessary to study the contribution of Brd2 and Brd3 to the regulation of the cellular transcriptome by JQ1.

Our analysis strongly supports the model that Brd4 is the key mediator of the therapeutic effects of JQ1 and that Brd4 not only represses but also induces many genes. Besides cell proliferation, Brd4 inhibition affects cellular function quite globally, possibly including the activity of cell type-specific super-enhancers^10^. Given the functional dependence of Brd4 on its P-TEFb interacting domain, new inhibitors should be developed to target this interaction.

## Materials and Methods

### Plasmids and cell culture

HA-tagged Brd4 constructs were cloned into the pRTS-1 vector as previously described^19, 42^. Briefly, using primers that provided a CCACC Kozak sequence followed by a start codon (Supplementary Materials and Methods), the open reading frame (ORF) of *BRD4*, or subfragments of the ORF (designated f1-f9), were amplified by PCR with a pFLAG-CMV2-BRD4 (1–1362) plasmid as template DNA (Addgene #22304)^34^. A SV40 nuclear localization signal (PKKKRKV) was added using designed reverse primers. C-terminal HA tag, STOP codon and two *SfiI* restriction sites that flanked the whole ORF were added via blunt-end ligation of the PCR product with sub-cloning construct pSfiExpress. After *SfiI* restriction digest the fragment was ligated with the pRTS-1 vector, which features a doxycycline-inducible bidirectional promoter, also expressing eGFP. Expression cassettes of all final plasmids were verified via sequencing. A pRTS-1 construct containing the luciferase gene served as vector control.

Identity of Raji cells (DSMZ no.: ACC 319) was verified by determining genetic characteristics using PCR-single-locus-technology (Eurofins Genomics). Raji and H1299 cells were stably transfected using PolyFect (Qiagen) and selected for 1 week in the presence of 100 µM hygromycin B (Amiresco), which was increased to 200 µM for 3 additional weeks. Expression of recombinant proteins was induced by adding 25 ng/mL or 1000 ng/mL doxycycline (Sigma-Aldrich) to Raji or H1299 cell culture medium, respectively. These concentrations had to be determined for each cell line individually, because doxycycline itself caused proliferation defects when used at high concentrations. Cells were cultured in RPMI (Raji) or DMEM (H1299) (Gibco, Life Technologies) supplemented with 10% fetal calf serum, 2 mM L-glutamine (Gibco, Life Technologies), and 1% penicillin streptomycin (Gibco, Life Technologies) at 37 °C, 5% CO_2_ (Raji) or 8% CO_2_ (H1299).

### Cell proliferation assays

Cell culture density was measured using different systems. Proliferation of H1299 cells was monitored via the xCELLigence system (Roche). Because the xCELLigence systeme cannot be applied to suspension cells, a Vi-CELL XR cell counter (Beckman Coulter) was used to measure proliferation of Raji cells transfected with Brd4 constructs. The long-term impact of JQ1 on Raji cells was detected with a Countess automated cell counter (Invitrogen, Thermo Fisher Scientific).

Cell proliferation at increasing JQ1 concentrations was assessed using the CellTiter 96 AQueous One Solution Cell Proliferation Assay System (Promega). Cells were seeded in a 96-well plate and increasing concentrations of JQ1 or DMSO (control) were added. After 72 h MTS tetrazolium compound was added to each well for one hour. Then the quantity of the MTS formazan product was measured as absorbance at 490 nm with a Sunrise photometer (TECAN) which was operated using the Magellan data analysis software (v7.2, TECAN). Relative signals were calculated by dividing the JQ1 signals by the corresponding DMSO signals.

### Flow cytometry

The eGFP-positive cell population was quantified by means of flow cytometry. Cells were washed, resuspended in phosphate buffered saline, and submitted to an XCalibur flow cytometer (BD Biosciences). For data acquisition, non-induced Raji cells were gated for lymphocytes from which the eGFP-negative gate was defined for the following measurements. FlowJo 2 Software (v.10.0.8, FlowJo, LLC) was used for data analysis.

### Western blot and antibodies

Cells were lysed in 2x laemmli buffer, boiled at 95 °C for 5 min, and sonified (Sonifier 250 [Branson], 10 pulses, output 5, duty cycle 50%) before submission to SDS-PAGE and transfer to a nitrocellulose membrane (GE Healthcare). Prior to primary antibody incubation, unspecific binding of antibodies was blocked by incubation of the membrane with 5% milk in Tris-buffered saline (1% Tween). Primary antibodies were used as follows: anti-HA (3F10, Roche) and anti-α-tubulin (T6557, Sigma). Afterwards the membrane was incubated with HRP-conjugated secondary rat (Jackson Laboratories) or mouse (Promega) specific antibodies and the signals were visualized via enhanced chemiluminescence using the Amersham ECL Western Blotting Detection Reagent (GE healthcare).

### Library preparation

24 hours after induction/JQ1 treatment, cells were washed twice in DPBS (Gibco, Life Technologies), resuspended in TRIzol reagent (Ambion, Life Technologies) and short-term stored at −80 °C until library preparation. Total RNA was isolated using the Direct-zol kit (Zymo research). The quality of total RNA was controlled using a nanodrop ND1000 photospectrometer (Thermo Scientific): The ratios of absorbance at 260 nm and 280 nm ranged from 1.93 to 1.97 and 260/230 ratios ranged from 2.1 to 2.2. RNA integrity was checked on an Agilent BioAnalyzer 2100 with the RNA nano chip kit (Agilent Technologies): RNA integrity numbers (RIN) ranged from 9.7 to 10 (scale 1 to 10; 10 for highest quality).

Next 1 µg of total RNA was used for preparation of Illumina-compatible strand-specific cDNA (RNA-seq) libraries using the mRNA-SENSE kit from Lexogen (Vienna). Briefly, polyA RNA was bound to oligo dT beads; starter and stopper heterodimer oligos were annealed to captured mRNA. Starter oligos served as primer for cDNA Synthesis which continued until stopper. Stopped cDNA and stopper were ligated to yield a cDNA fragment flanked by Illumina P5 and P7 sequences. This was followed by PCR amplification, which introduced barcodes and adaptor sequences to the libraries. PCR products were purified with Ampure XP beads (Beckman-Coulter), followed by quantification and quality control with Agilent BioAnalyzer 2100 using a DNA 1000 chip. Samples were sequenced on Illumina HiSeq 1500 (Illumina Inc.) in single end mode with 76 bp read length and i7 index read. The sequencing depth was ~2 × 10^7^ reads per sample.

### Bioinformatic analysis

After trimming adapters from sequencing reads using cutadapt^43^, reads were mapped to the human reference genome GRCh38 as well as rRNA sequences using ContextMap 2^44^. Read counts per gene were determined using featureCounts^45^ on the Ensembl gene annotation (release 84). Reads were assigned to a gene if they overlapped with an exon of the gene by at least 25 bp and mapped uniquely to that gene. ~80% of reads could be mapped per sample. Genes were included in downstream analyses if the average read count across all samples was ≥25 (~11,700 genes).

Before gene expression analysis, principal component analysis (PCA) and hierarchical clustering of samples were performed. For PCA, read counts were normalized using limma^46^. Hierarchical clustering analysis was based on the Euclidean distances between the normalized read counts. Differential expression analysis for each condition against the control was performed with limma, edgeR and DESeq2^47, 48^. Multiple testing correction was performed using the Benjamini–Hochberg method^49^. Genes were selected for further analysis if they were identified as differentially expressed by at least two of the three methods (multiple testing corrected p-value ≤ 0.05, Supplementary Table 1). GSEA^50^ was performed using lists of genes that were ranked according to log2 fold-changes obtained from the previous DE analysis. C-Myc target gene sets were collected from the Molecular Signatures Database^51^.

## Electronic supplementary material

Supplementary Info

Supplementary Table 1
