# Supplementary material for: Transcriptome analysis of dominant-negative Brd4 mutants identifies Brd4-specific target genes of small molecule inhibitor JQ1
Source: Sci Rep. 2017 May 10;7:1684. doi: 10.1038/s41598-017-01943-6 (PMC5431861; doi:10.1038/s41598-017-01943-6)
Supplement: Supplementary file 1 — Supplementary Info [file 41598_2017_1943_MOESM1_ESM.pdf]

Supplementary information

Transcriptome analysis of dominant-negative Brd4 mutants identifies Brd4-specific target genes of small molecule inhibitor JQ1

Tim-Michael Decker[1+], Michael Kluge[2+], Stefan Krebs[3], Nilay Shah[1], Helmut Blum[3], Caroline C Friedel[2], Dirk Eick[1]*

[1]Department of Molecular Epigenetics, Helmholtz Center Munich and Center for Integrated Protein Science Munich (CIPSM), Marchioninistrasse 25, 81377 Munich, Germany. [2]Institute for Informatics, Ludwig-Maximilians-Universität München, Amalienstr. 17, Munich, 80333, Germany. [3]Laboratory for Functional Genome Analysis (LAFUGA) at the Gene Center, Ludwig-Maximilians-Universität München, Munich, Germany.

*Correspondence should be addressed to DE (email: eick@helmholtz-muenchen.de).

[+]These authors contributed equally to this study.

Supplementary material

Oligonucleotides

wt fwd	CCACCATGTCTGCGGAGAGCG
frag2 fwd	CCACCATGATGATAGTCCAGGCAAAAG
frag3 fwd	CCACCATGACCACCATTGACCCCATTC
frag4 fwd	CCACCATGGTCCGATTGATGTTCTCCAAC
frag5 fwd	CCACCATGAAGCACAAAAGGAAAGAGGAAGTGG
frag6 fwd	CCACCATGCACCATCATCACCACCATCAG
frag7 fwd	CCACCATGACCCAAACACCCCTGCTC
frag8 fwd	CCACCATGCACAAGTCGGACCCCTAC
frag9 fwd3	CCACCATGGCCCCTGACAAGGACAAA
wt rev	GAAAAGATTTTCTTCAAATATTGAC
frag1_NLS rev	TACCTTTCTCTTCTTTTTTGGCTGGGTCTGCGGAGGAG
frag2_NLS rev	TACCTTTCTCTTCTTTTTTGGCTTCTTGGCAAACATCTC
frag3_NLS rev	TACCTTTCTCTTCTTTTTTGGGCTATCGCTGCTGCTGTC
frag4_NLS rev	TACCTTTCTCTTCTTTTTTGGCTTGTCCTCTTCCTCCGAC
frag5_NLS rev	TACCTTTCTCTTCTTTTTTGGAATGAAGGGTGGGGGCGAGG
frag6_NLS rev	TACCTTTCTCTTCTTTTTTGGGGAAGGGAGTAGCGGCGT
frag7_NLS rev2	TACCTTTCTCTTCTTTTTTGGCTCCTTCACCACCACAAG
frag8_NLS rev	TACCTTTCTCTTCTTTTTTGGCTTCTCACGCTCCTCTTTC
wt_NLS rev	TACCTTTCTCTTCTTTTTTGGGAAAAGATTTTCTTCAAATATTGAC

Supplementary Table S1*. Differential expression data of significantly regulated genes. Programs limma, edgeR and DEseq2 were used to perform differential expression analysis of conditions JQ1, f3 and f9 relative to the Raji-luc DMSO control. Genes were considered as differentially expressed genes, if they were identified as such by at least two of the three methods (multiple testing corrected p-value ≤0.05).

* This table was attached as file ‘supp_table1.xls‘.

Supplementary Table S2. Differential expression analysis of representative genes. Representative genes were listed according to the median log2 fold changes (FC) determined for the JQ1 samples. Median log2 FCs were calculated using the log 2 FC values determined by the differential expression programs that found a statistical significant change (at least 2 out of limma, edgeR, DEseq2). Upregulated genes are labeled in blue, downregulated genes are labeled in green. NS (not significant). (a) *C-Myc* and *c-Myc*-regulated genes. (b) Genes encoding for subunits of the super elongation complex (SEC).

a

	median log2 FC		
**Gene**	**f3**	**f9**	**JQ1**
CDKN1B	0.67	1.09	1.16
CDKN2B	1.89	1.88	1.53
c-Myc	-1.14	-0.86	-1.21

induced

repressed

b

		median log2 FC		
	**Gene**	**f3**	**f9**	**JQ1**
**super elongation complex (SEC)**	AFF1	1.07	0.67	1.12
	AFF2	0.70	NS	1.10
	AFF4	0.67	0.45	0.95
	MLLT3 (AF9)	0.99	0.75	0.92
	CDK9	NS	NS	0.34
	EAF1	-0.23	NS	NS
	MLLT1 (ENL)	-0.27	-0.28	-0.41
	EAF2	NS	NS	-0.60
	AFF3	NS	-0.43	-1.27

induced

repressed

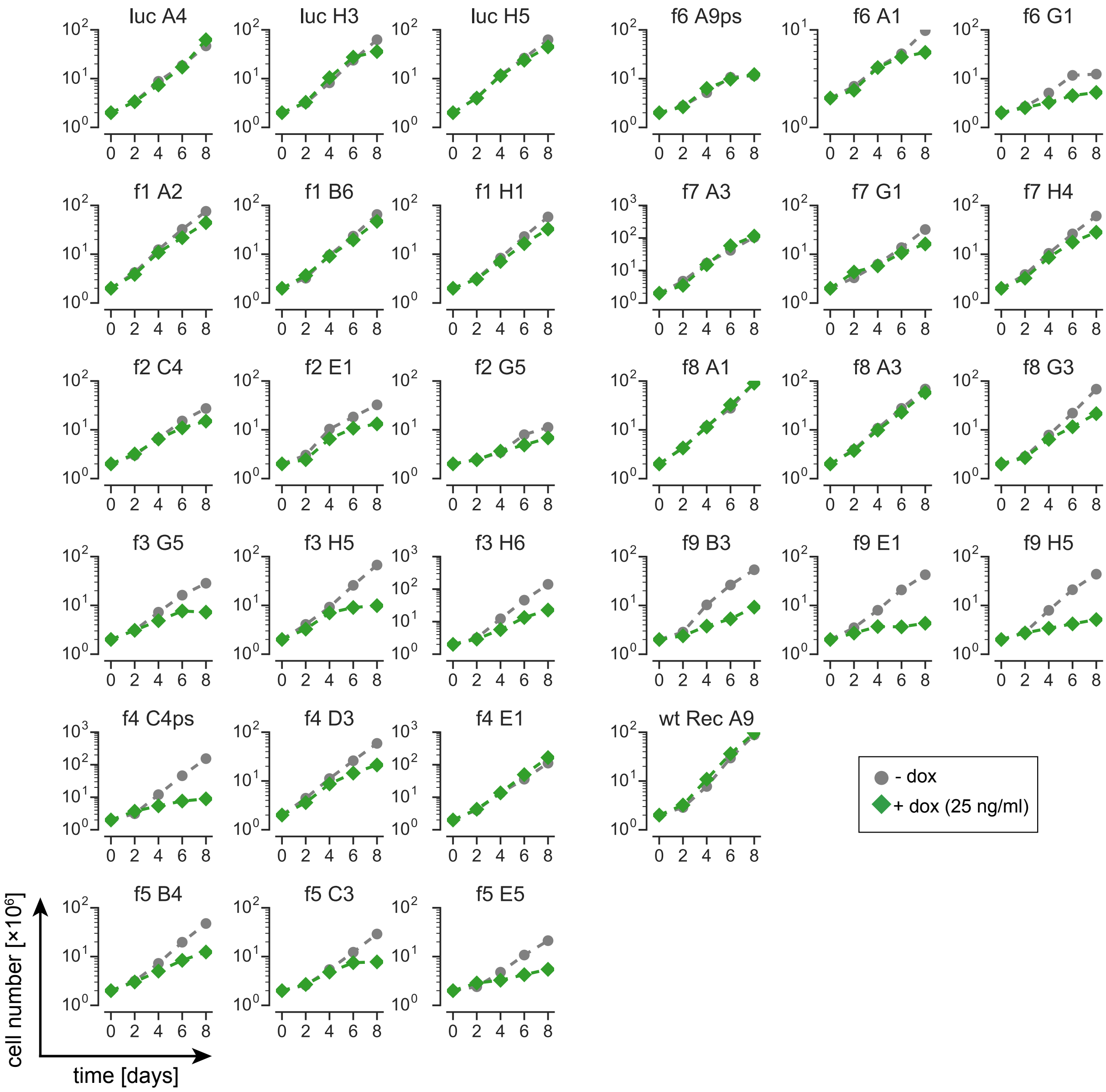

Supplementary Figure S1. Proliferation assay for Brd4 mutants. Living cell numbers (log scale) of induced (25 ng/ml doxycycline, green) vs. non-induced (gray) cells were plotted against time (days). Induced cells expressed either luciferase (Raji-luc, negative control), Brd4 fragments f1 - f9 or recombinant full-length Brd4 (wt Rec). For each condition three biological replicates were analyzed, except wt Rec, for which we obtained only one inducible clone. Mutant f4 showed inconsistent proliferation defects, because the mutant was not inducible in one of the three biological replicates. Proper induction of all constructs was controlled by measuring GFP-positive cells using flow cytometry (Supplementary Fig. S2). This revealed that f4-E1 was not induced on day 8 indicating that the dominant-negative conditions selected for a non-inducible subpopulation in this replicate.

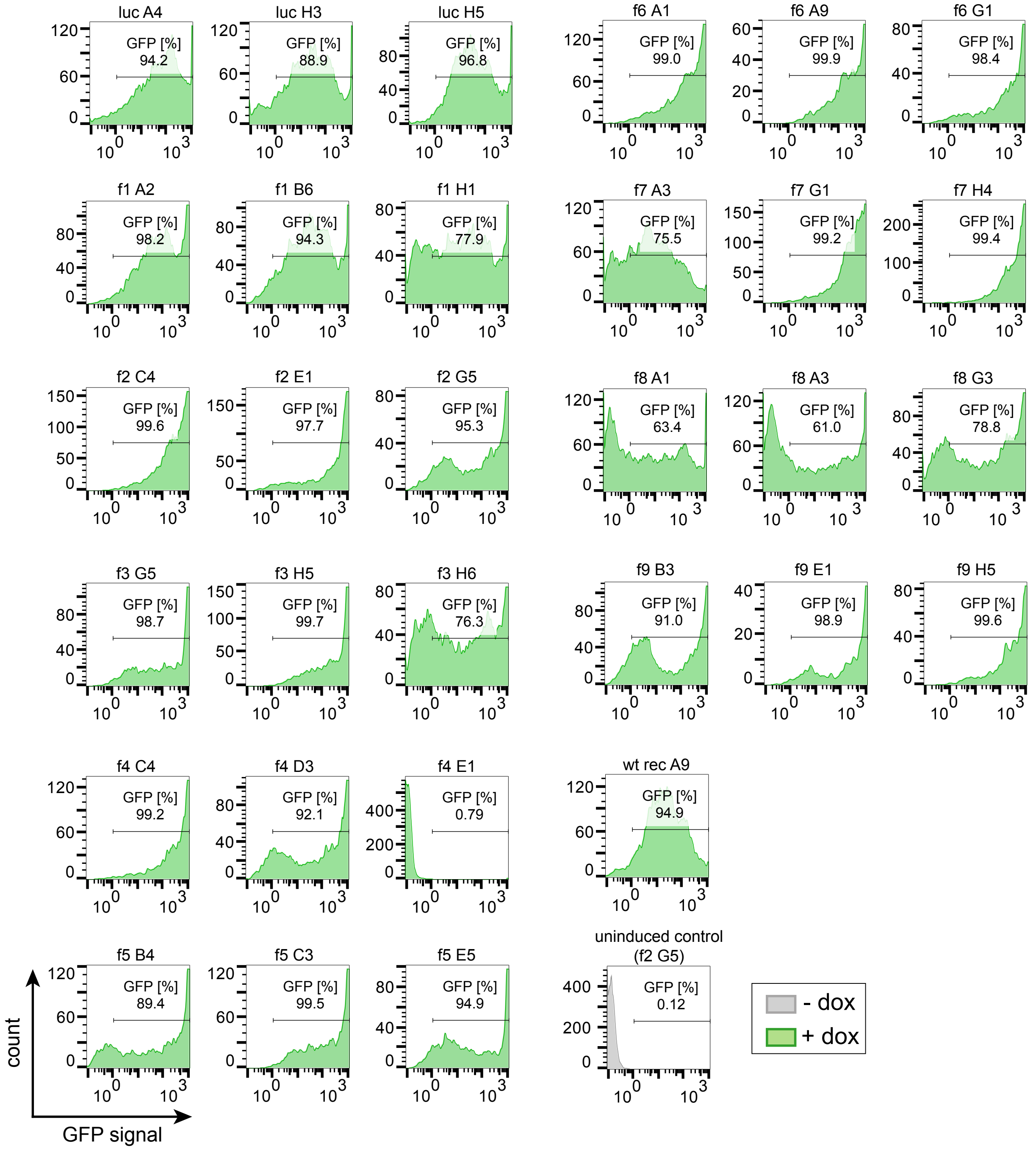

Supplementary Figure S2. Induction of Brd4 mutants used in the proliferation assay. Ratios of induced cells (green) were assessed by measuring GFP reporter signals using flow cytometry as a control for the proliferation assay on day 8 (Supplementary Fig. S1). Uninduced Raji f2 cells served as negative control (gray). For most replicates induction rates of 90% or higher were measured. For f4 E1 a non-inducible subclone was selected.

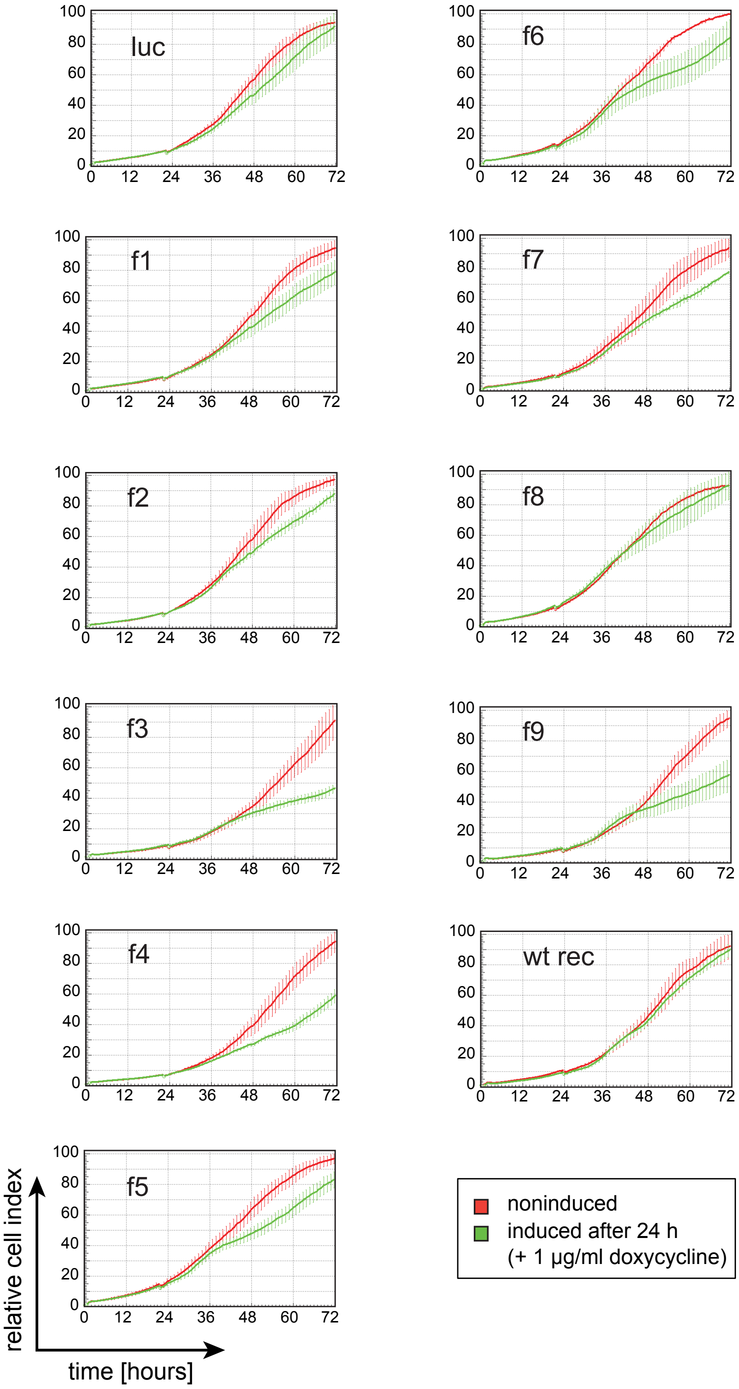

Supplementary Figure S3. Proliferation assay for dominant-negative Brd4 mutants in H1299 cells. Proliferation of H1299 cells that express Brd4 constructs or luciferase (control) was measured using the XCelligence system (Roche). Expression of the recombinant proteins was induced via 1 µg/ml doxycycline 24 h after seeding of the cells. The cell index of induced (green) and non-induced (red) cells was measured for additional 48 h and plotted against time. Error bars: standard deviation, n=2.

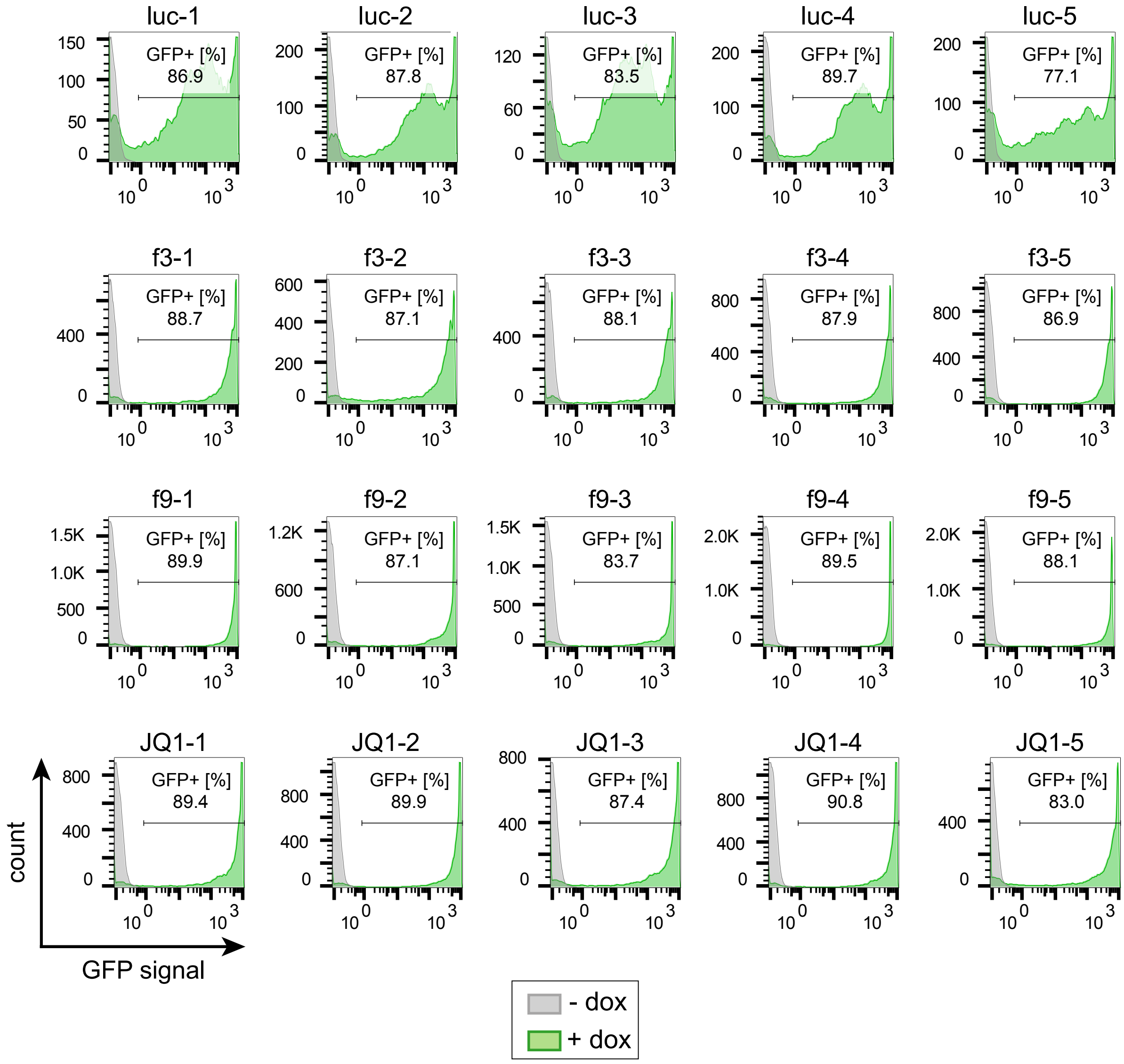

Supplementary Figure S4. Induction of Brd4 mutants used for RNA-seq. Ratios of induced cells (green) vs. non-induced cells (grey) were assessed by measuring eGFP reporter signals using flow cytometry.

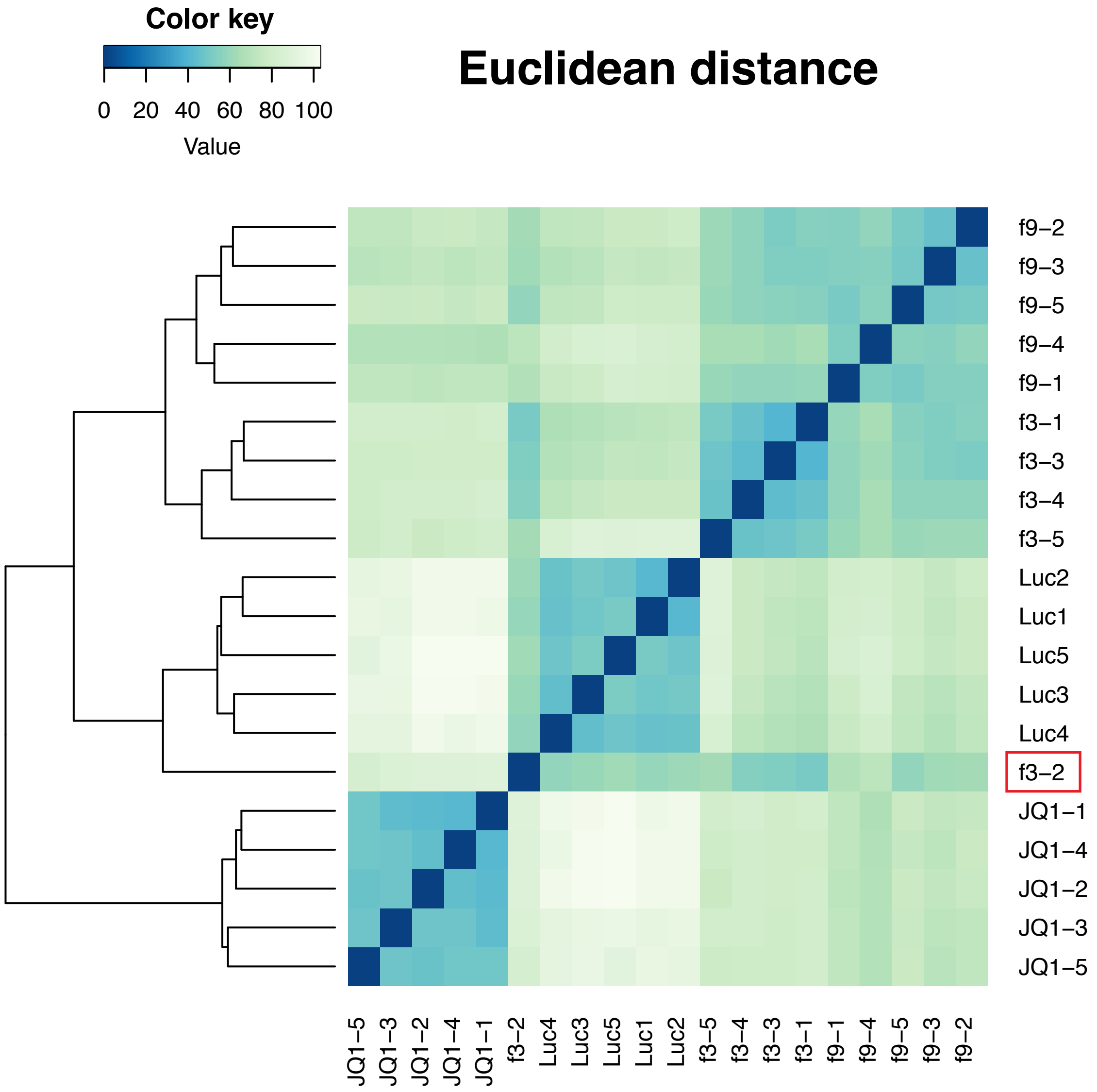

Supplementary Figure S5: Hierarchical clustering analysis. Clustering analysis was based on the Euclidean distances between the normalized read counts. Replicate f3-2 (marked in red) clustered near to the control group and was excluded from further analyses.

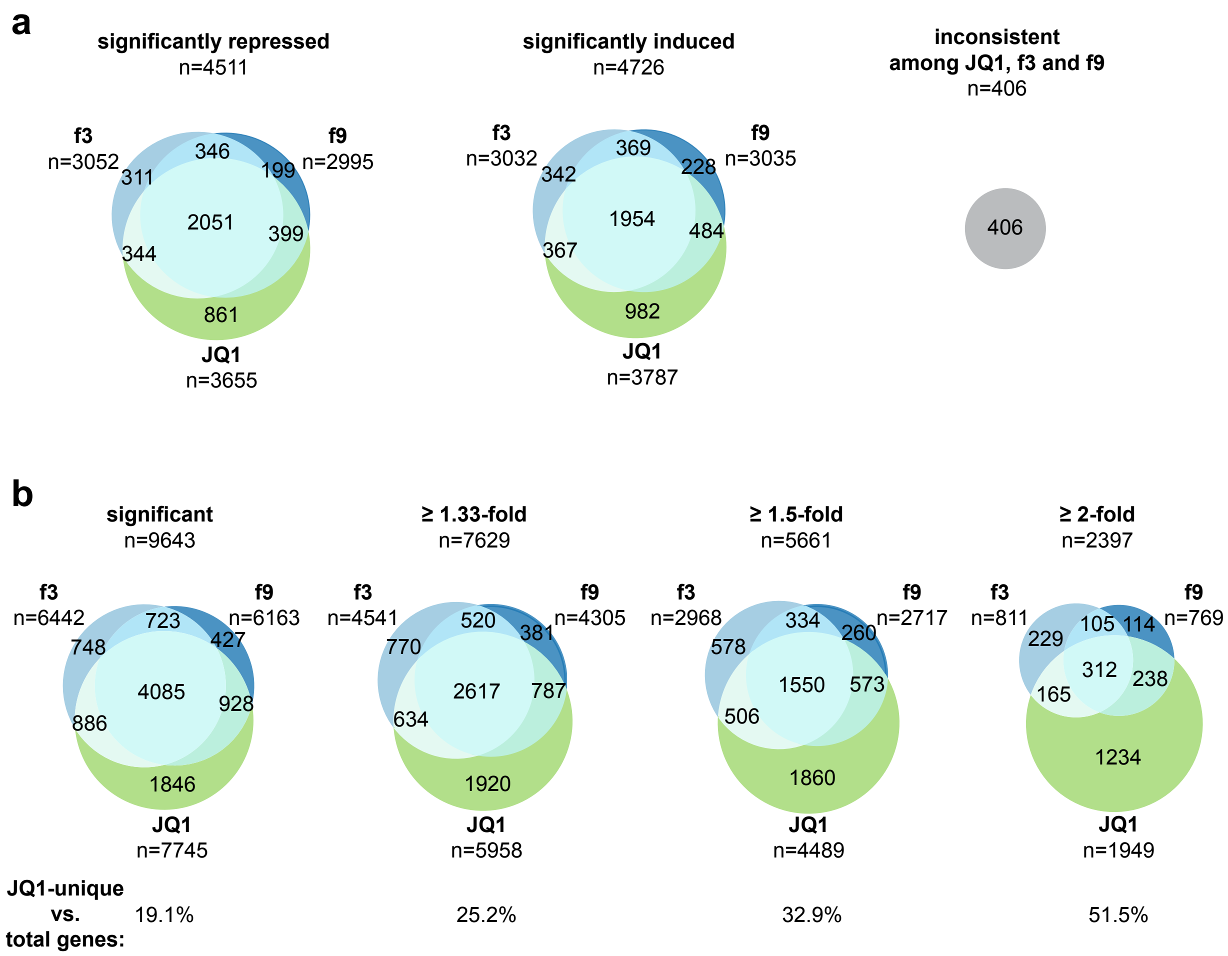

Supplementary Figure S6. Differential gene expression induced by JQ1 and dnBrd4 mutants f3 and f9. Differential expression analysis was performed using three programs: limma, edgeR and DESeq2. Genes that were found significantly (p-value ≤0.05) differentially expressed with at least 2 out of the 3 programms (DE genes) were used for further analysis. (a) Genes were split into repressed (left, 4511), induced (middle, 4726) and inconsistent genes (right, 406). A gene was considered as inconsistently regulated when it was upregulated in one group and downregulated in another group. Overlapping gene sets between f3, f9 and JQ1 datasets were visualized in Venn diagramms. (b) DE genes were filtered by applying increasing fold-change filters. The percentage of JQ1-unique genes (green) relative to the total number of DE genes that passed the filter was calculated. Notably, most of the 2-fold filtered JQ1-unique genes (right) were found as genes regulated in mutant f3 or f9, or in both when significantly regulated genes were examined (left).
